# Ferroptosis: The Demise of Cells Through Phospholipid Peroxidation

**DOI:** 10.1002/advs.202524387

**Published:** 2026-03-20

**Authors:** Shaojie Cui, Jin Ye

**Affiliations:** ^1^ College of Biomedicine and Health College of Life Science and Technology Huazhong Agricultural University Wuhan Hubei China; ^2^ Department of Molecular Genetics University of Texas Southwestern Medical Center Dallas Texas USA

**Keywords:** ferroptosis, GPX4‐glutathione defense, hyperoxidized PRDX3, phospholipid peroxidation, PUFA‐containing phospholipids

## Abstract

Ferroptosis is a form of regulated cell death driven by iron‐dependent peroxidation of polyunsaturated phospholipids. The susceptibility of cells to ferroptosis is often strongly shaped by lipid metabolic pathways that determine the abundance, distribution, and redox reactivity of polyunsaturated phospholipids. Enzymes that activate and incorporate polyunsaturated fatty acids into phospholipids generate the substrates whose peroxidation causes ferroptosis, thereby sensitizing cells to ferroptosis. In contrast, synthesis of monounsaturated phospholipids and the presence of lipid‐soluble antioxidants limit the accumulation of phospholipid peroxides, thereby protecting cells from ferroptosis. Thus, different tissues may display characteristic ferroptotic responses caused by their unique lipid composition and antioxidant capacity. This review summarizes the metabolic foundations that determine the susceptibility of cells to ferroptosis and discusses the possibility of treating human diseases by modulating cellular sensitivity to ferroptosis.

## Graphical abstract

The relative abundance of peroxidation‐resistant monounsaturated fatty acids (MUFAs) vs. peroxidation‐sensitive polyunsaturated fatty acids (PUFAs) incorporated into phospholipid (PL) is a key determinant of ferroptosis sensitivity, as the peroxidation of PL PUFA acyl chains (PUFA‐PL) disrupts membrane integrity, causing ferroptotic cell death.

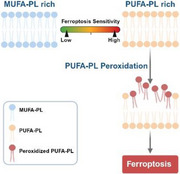

## 1 Introduction: Ferroptosis Depends on Lipid Metabolism

Ferroptosis is a form of regulated cell death driven by iron‐dependent lipid peroxidation. Unlike other cell death pathways, such as apoptosis or necroptosis, ferroptosis is not executed through a particular protein effector; instead, it reflects a membrane‐centered redox catastrophe in which chain‐propagating lipid peroxidation outpaces cellular detoxification capacity [1, 2]. The glutathione peroxidase 4 (GPX4)–glutathione (GSH) system that reduces phospholipid (PL) hydroperoxides is the classic defense mechanism against ferroptosis. The failure of this system allows accumulation of PL hydroperoxides, which compromise membrane integrity and cellular viability [3, 4]. In addition to GPX4, other systems can intercept lipid peroxidation in defined subcellular contexts, thereby raising the threshold for ferroptosis. For example, ferroptosis suppressor protein 1 (FSP1) regenerates reduced coenzyme Q (CoQ) and vitamin K, lipid‐soluble antioxidants that terminate the chain reaction of lipid peroxidation, thereby protecting cells from ferroptosis, particularly under the conditions of GPX4 insufficiency [5, 6]. The mitochondrial dihydroorotate dehydrogenase (DHODH)–CoQ and the GTP cyclohydrolase 1 (GCH1)–tetrahydrobiopterin (BH4) pathways also protect cells from ferroptosis through parallel mechanisms that inhibit lipid peroxidation [7, 8, 9]. Conversely, oxidoreductases such as cytochrome P450 oxidoreductase (POR) and cytochrome b5 reductase 1 (CYB5R1), which promote lipid peroxidation through their enzymatic activity, sensitize cells to ferroptosis [10, 11, 12]. Thus, rather than generic oxidative‐stress, ferroptosis is better viewed as failure to relieve lipid‐peroxidation pressure by various anti‐peroxidation defenses [13, 14].

Accumulating evidence supports a lipid‐centric framework for ferroptosis susceptibility. In addition to iron and oxidative stresses, the necessary components of ferroptosis, vulnerability of cells to ferroptosis is often strongly shaped by the availability, distribution, and redox reactivity of peroxidizable membrane substrates, that is, the balance between polyunsaturated fatty acid (PUFA) ‐containing PLs and the regenerative capacity of anti‐peroxidation defenses. Here, we adopt this membrane‐centric perspective as a framework to emphasize how lipid composition interacts with iron and redox biology to set the threshold for ferroptosis.

In this Review, we discuss how the biosynthesis and remodeling of PUFA‐ and monounsaturated fatty acid (MUFA)‐containing PLs shape ferroptosis sensitivity, how ferroptotic death is executed through PL peroxidation, and how tissue‐specific metabolic contexts influence ferroptotic vulnerability. We further discuss translational opportunities and limitations in modulating ferroptosis in vivo, including biomarker‐guided detection and context‐dependent intervention strategies.

## 2 Protection Against Ferroptosis by Maintaining Homeostasis of PUFA‐PLs

The execution of ferroptosis depends on the presence of PUFA‐PLs within cellular membranes [1, 15, 16]. These PUFA acyl chains, such as arachidonic acid (AA, 20:4) and docosahexaenoic acid (DHA, 22:6), are susceptible to peroxidation due to their bis‐allylic C─H bonds [17, 18]. Thus, in many experimental systems, PUFA‐PL levels are a major determinant of ferroptosis sensitivity [15, 16]. Mammalian cells counter this liability by regulating lipid metabolic fluxes to constrain excessive accumulation of PUFA‐PLs and/or by increasing MUFA‐PLs that resist lipid peroxidation.

### 2.1 Regulating PUFA‐PL Synthesis to Protect Cells from Ferroptosis

Mammalian cells lack the enzyme that converts MUFAs to PUFAs. Thus, linoleic acid (LA, 18:2) and α‐linolenic acid (ALA, 18:3), the shortest ω‐6 and ω‐3 PUFAs, respectively, are considered essential fatty acids that must be acquired through the diet [19]. LA and ALA can be converted to AA and DHA, respectively, in mammalian cells, through sequential elongation and desaturation catalyzed by ELOVL (elongation of very‐long‐chain fatty acids) and FADS (fatty‐acid desaturase) enzymes [19, 20].

**FIGURE 1:**
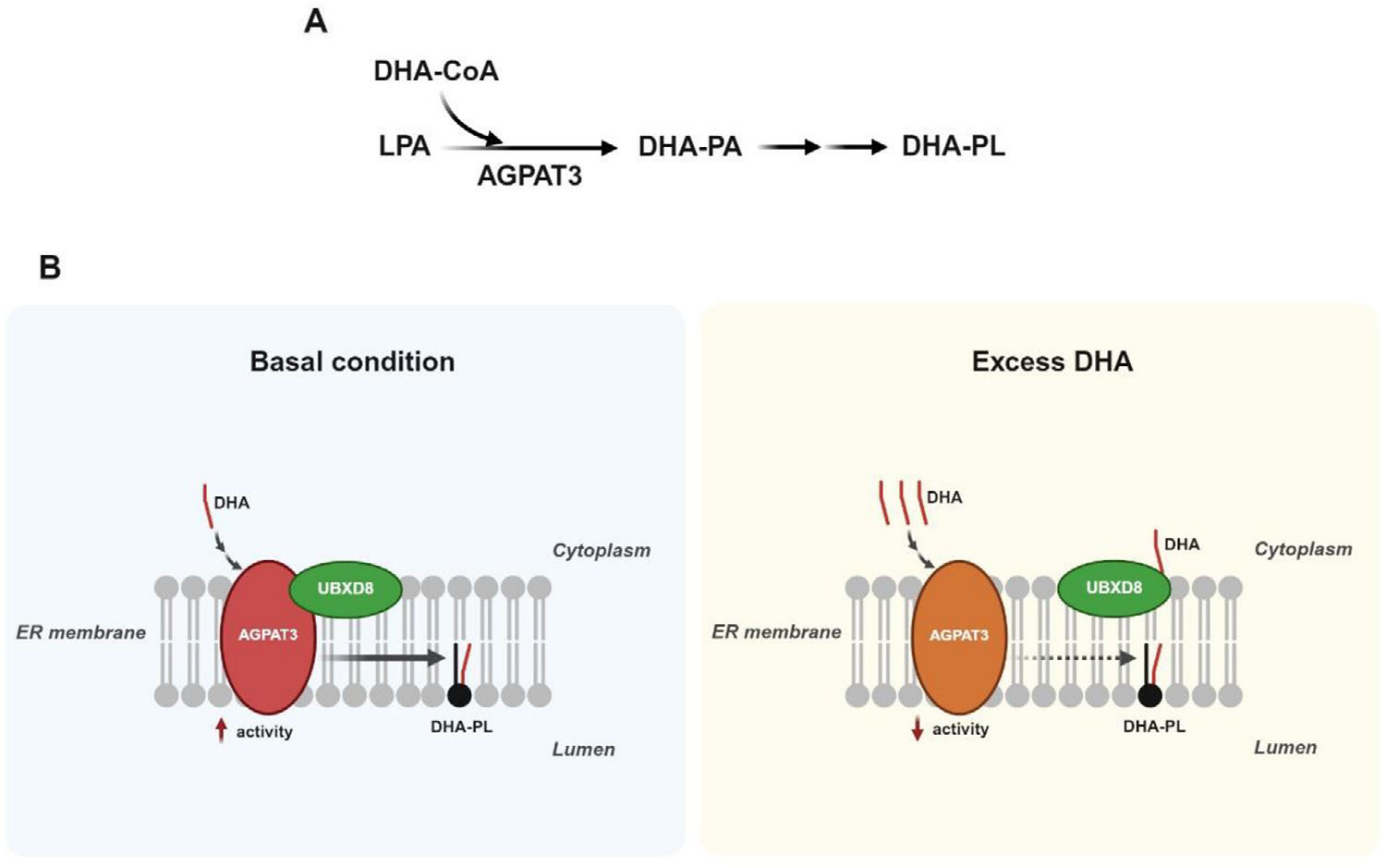
Synthesis and feedback regulation of DHA‐containing PLs. (A) In mammalian cells, DHA is incorporated into membrane PLs primarily through de novo synthesis via the Kennedy pathway. AGPAT3 catalyzes the incorporation of DHA‐CoA into lysophosphatidic acid (LPA) to generate DHA‐containing phosphatidic acid (PA), which is subsequently converted into DHA‐containing PLs (DHA‐PLs) through multiple reactions. (B) A feedback mechanism maintains DHA‐PL homeostasis by sensing excess DHA. Under basal conditions, UBXD8 forms a complex with AGPAT3 and promotes its activity to sustain adequate synthesis of DHA‐PLs. When cells are exposed to excess DHA, DHA binds to UBXD8 and disrupts the UBXD8‐AGPAT3 complex, thereby inhibiting AGPAT3 activity and preventing overproduction of DHA‐PLs. This feedback regulation helps maintain DHA‐PL homeostasis to protect cells from ferroptosis. This figure was created using BioRender (https://biorender.com/).

However, free PUFAs alone are often insufficient to induce ferroptosis. Their incorporation into PLs is also required to efficiently support the propagation and execution of ferroptosis [15]. The composition of PUFA‐PL pools is determined by the amounts PUFAs obtained exogenously through dietary intake and those synthesized endogenously through elongation/desaturation, and different PUFAs may be incorporated into PLs through different reactions. In mammalian cells, DHA is primarily incorporated into membrane PLs through de novo synthesis known as the Kennedy pathway [21]. In this pathway, 1‐acylglycerol‐3‐phosphate O‐acyltransferase 3 (AGPAT3) catalyzes the incorporation of DHA‐CoA into lysophosphatidic acids (LPAs) to produce DHA‐containing phosphatidic acid (PAs) [22, 23, 24], which, through two following reactions, are converted to DHA‐containing PLs (Figure 1A). Interestingly, mammalian cells are able to sense the presence of DHA to regulate AGPAT3 activity. In the absence of excess DHA, AGPAT3 binds to ubiquitin regulatory X domain‐containing protein 8 (UBXD8), and this interaction leads to AGPAT3 activation so that adequate synthesis of DHA‐PLs is maintained (Figure 1B) [24]. Excess DHA, through its interaction with UBXD8 [25], disrupts the UBXD8‐AGPAT3 complex. In the absence of UBXD8‐mediated activation, the activity of AGPAT3 is inhibited, thus preventing overproduction of DHA‐PLs in cells exposed to excess DHA, a reaction protecting them from ferroptosis (Figure 1B) [24].

**FIGURE 2:**
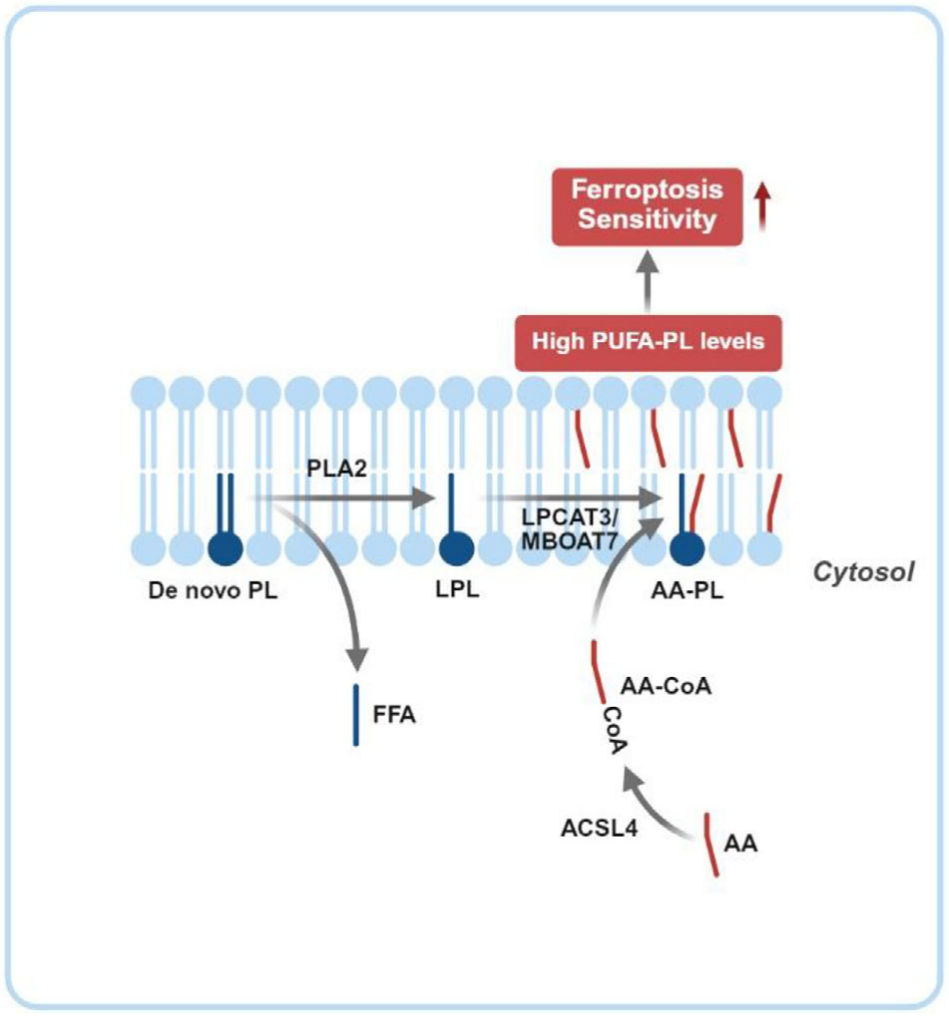
Generation of AA‐containing PLs through PL remodeling. In mammalian cells, AA‐containing PLs (AA‐PLs), a major class of PUFA‐PLs, are generated through the PL remodeling pathway known as the Lands’ cycle. In the first step, phospholipase A2 (PLA2) family enzymes cleave newly synthesized PLs to generate lysophospholipids (LPLs) and free fatty acid (FFA). In the second step, AA is activated to AA‐CoA primarily by ACSL4, and then re‐esterified into LPLs by LPLATs, such as LPCAT3 or MBOAT7, to produce AA‐PLs, which sensitize cells to ferroptosis. This figure was created using BioRender (https://biorender.com/).

In contrast to DHA, AA is primarily incorporated into PLs through the PL remodeling pathway known as the Lands’ cycle [26, 27]. In the first step of this pathway, newly synthesized PLs are cleaved by phospholipase A2 (PLA2) family enzymes to generate lysophospholipids (LPLs). The Ca^2+^‐independent phospholipase A2 (iPLA2) family of the phospholipase has been proposed to be involved in this reaction to maintain PL homeostasis [28], as treatment with a pan‐iPLA2 inhibitor blocked synthesis of AA‐PLs [29]. In the second step, LPL acyltransferases (LPLATs) with substrate specificity tailored to AA, such as lysophosphatidylcholine acyltransferase 3 (LPCAT3) or membrane bound *O*‐acyltransferase 7 (MBOAT7) [30, 31], catalyze acylation of the LPLs with AA‐CoA generated primarily by long chain acyl‐CoA synthetase 4 (ACSL4) [15] to produce AA‐PLs (Figure 2). Consistent with observations that ferroptosis sensitivity is determined by PUFA‐PL levels, cells deficient in LPCAT3 or ACSL4 are more resistant to ferroptosis owing to a deficiency in the synthesis of AA‐PLs [15, 30]. It was reported that excess AA triggered proteasomal degradation of ACSL4 [32], and LPCAT3 was stabilized by choline/ethanolamine phosphotransferase 1 (CEPT1), an enzyme involved in PC and PE synthesis [33]. It remains to be determined whether these regulations, in a way analogous to the regulation of DHA‐PL synthesis, also prevent overproduction of AA‐containing PLs.

Somewhat unexpectedly, knocking out iPLA2β, one of the iPLA2s, actually sensitized cells to ferroptosis [34, 35]. A plausible explanation is that, in addition to Lands’ cycle, iPLA2β is also active in removing peroxidized acyl chains from PLs [34]. While other iPLA2s may compensate for the loss of iPLA2β in generating AA‐PLs through the Lands’ cycle, they may not be able to substitute iPLA2β to remove peroxidized PLs. Consequently, the loss of iPLA2β might impair this critical ferroptosis‐protecting activity without affecting the synthesis of AA‐PLs. In addition to iPLA2β, inhibiting lipoprotein‐associated phospholipase A2 (Lp‐PLA2), which belongs to a different family of PLA2, also sensitized cells to ferroptosis [36]. Interestingly, Lp‐PLA2 is also active in removing peroxidized PUFA chains from PLs [37]. Thus, besides GPX4‐catalyzed reduction of peroxidized PLs, the PLA2‐catalyzed hydrolysis of peroxidized PLs may also contribute to protection against ferroptosis.

### 2.2 Increasing MUFA‐PL/PUFA‐PL Ratio to Protect Cells from Ferroptosis

In addition to directly limiting PUFA‐PL synthesis, cells may reduce ferroptosis susceptibility by increasing the relative abundance of peroxidation‐resistant MUFA‐PLs. Human cells can generate MUFAs de novo through stearoyl‐CoA desaturases (SCDs), with SCD1 being a major isoform in most tissues and cancer cells [38]. Oleic acid (OA, 18:1), a MUFA, is incorporated into PLs through the same PL remodeling pathway discussed above. Inasmuch as OA competes with PUFAs for incorporation into the sn‐2 position of PLs, increasing OA‐PL synthesis limits production of PUFA‐PLs, thereby decreasing ferroptosis sensitivity [39, 40]. Indeed, among the multiple LPLATs that can use OA as a substrate, MBOAT1 and MBOAT2 have been reported to protect cells from ferroptosis [40].

Recent studies demonstrate that increasing MUFA‐PL synthesis is a strategy frequently used by tumor cells to evade ferroptosis by activating expression of SCD1, MBOAT1, or MBOAT2 [40, 41, 42, 43]. Thus, inhibiting MUFA‐PL synthesis has emerged as an attractive strategy to kill tumor cells through ferroptosis [44].

## 3 Execution of Ferroptosis by PL Peroxidation

The defining biochemical feature of ferroptosis is the excessive accumulation of lipid peroxides within cellular membranes [1, 3]. Lipid peroxidation is not a random oxidative event but a multistep chemical process propagated through radical chain reactions, ultimately causing cell death owing to the disruption of membrane integrity [45].

### 3.1 Mechanistic Basis of PL Peroxidation

**FIGURE 3:**
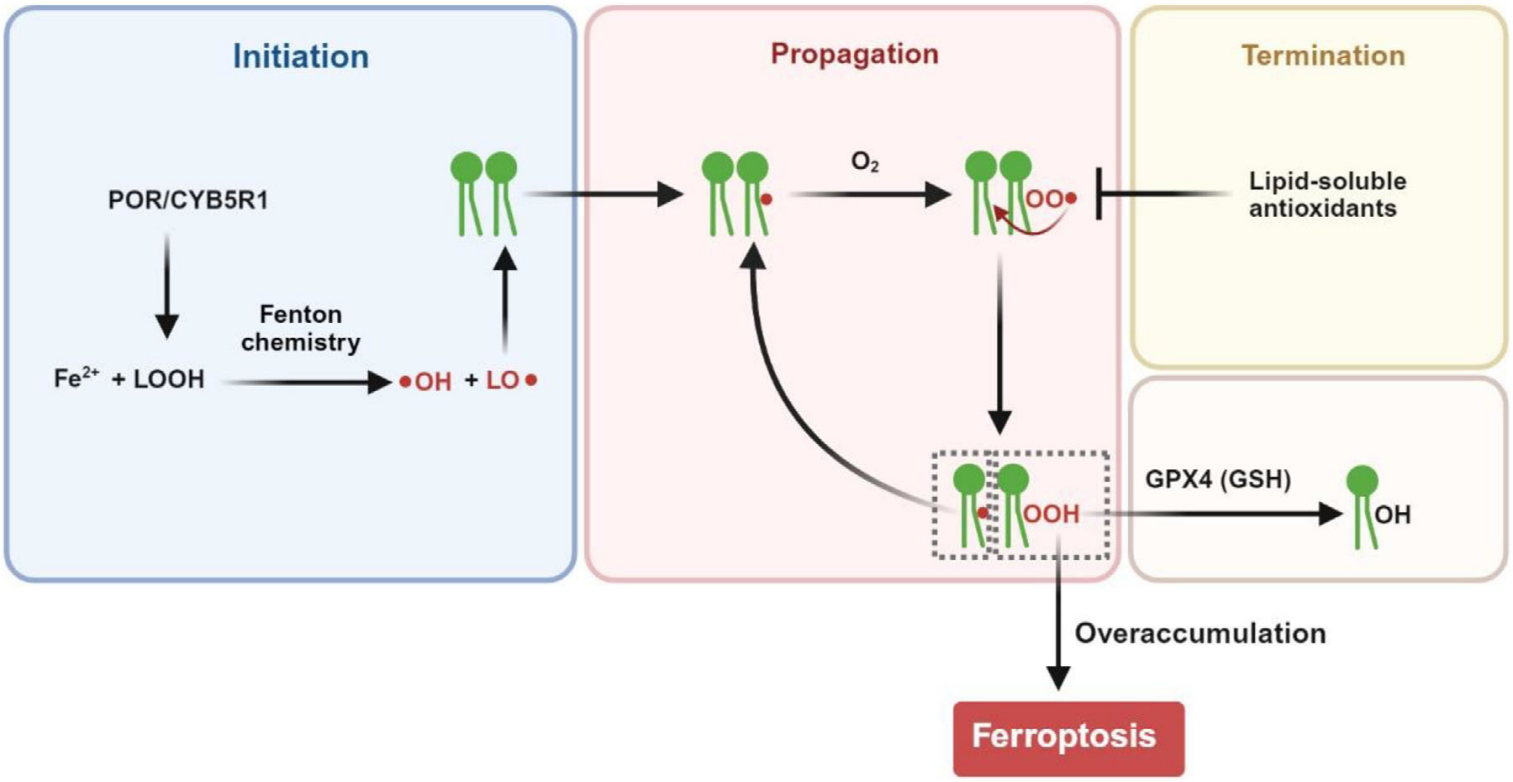
PL peroxidation drives ferroptotic cell death. Lipid peroxidation proceeds through three stages. During initiation, Fe2+ reacts with lipid hydroperoxides (LOOH) via Fenton chemistry to generate hydroxyl (•OH) and alkoxyl (LO•) radicals. In the propagation phase, LO• abstracts bis‐allylic hydrogen atoms from PUFA‐containing PLs (PUFA‐PLs), generating PL radicals (PL•), which react with oxygen to form peroxyl radicals (PLOO•). The PLOO• produced attack neighboring PUFA‐PLs to generate more PL•, thereby establishing a self‐amplifying chain reaction that leads to the accumulation of PL hydroperoxides (PLOOH). Lipid‐soluble antioxidants terminate PL peroxidation by neutralizing PLOO•. The GPX4‐GSH system detoxifies PLOOH by reducing PLOOH to PL alcohols (PLOH). When the rate of lipid peroxidation exceeds this detoxification capacity, excess PLOOH compromises membrane integrity and triggers ferroptotic cell death. The oxidoreductases POR and CYB5R1 are shown to promote LOOH production and thereby facilitating initiation of lipid peroxidation. This figure was created using BioRender (https://biorender.com/).

Lipid peroxidation follows a well‐defined chemical cascade involving three phases: initiation, propagation, and termination [45]. During initiation, Fe^2+^ reacts with lipid hydroperoxides (LOOH) through Fenton chemistry, producing highly reactive hydroxyl (•OH) and alkoxyl (LO•) radicals (Figure 3) [46, 47]. In the propagation phase, the alkoxyl radicals produced abstract hydrogen atoms from bis‐allylic positions of PUFA‐PLs, generating PL radicals (PL•), which rapidly react with molecular oxygen to form PL peroxyl radicals (PLOO•). The highly reactive PLOO• can then attack neighboring PUFA‐PLs, producing PL peroxides (PLOOH) and PL•, which react with oxygen again to establish a self‐amplifying oxidative cycle (Figure 3) [46, 47]. This chain reaction leads to the exponential accumulation of PLOOH, unless the PLOO• is neutralized by lipid‐soluble antioxidants such as vitamin E, vitamin K, CoQ, and 7‐dehydrocholesterol [5, 7, 48, 49] during the termination phase (Figure 3) [50]. To defend against overaccumulation of PLOOH, GPX4 reduces PLOOH to PL alcohols (PLOH) using glutathione (GSH) as a cofactor (Figure 3) [4, 51, 52]. When GPX4 is overwhelmed by the excess production of PLOOH, membrane integrity is compromised, triggering ferroptotic death. Thus, ferroptosis represents a biochemical imbalance where the rate of lipid peroxidation surpasses the capacity of detoxification [1, 3, 4].

In contrast to GPX4, certain enzyme‐catalyzed reactions can promote lipid peroxidation, thereby sensitizing cells to ferroptosis. For example, the reactions catalyzed by oxidoreductases POR and CYB5R1 may induce the generation of H_2_O_2_, which may stimulate production of LOOH that is fed into PL peroxidation, as shown in Figure 3 [10, 11, 12].

An intriguing question regarding ferroptosis is the identity of LOOH reacting with Fe^2+^ in the initiation phase that starts the whole process. They are unlikely to be the PLOOH themselves as PLs are located in membrane bilayers that are inaccessible to Fe^2+^ [45, 53]. One plausible candidate is free fatty acid peroxides (FFOOH). They are present in cytosol that could potentially react with Fe^2+^, and the LO• produced during the initiation phase are hydrophobic enough to enter PL membranes where they could drive PL peroxidation during the propagation phase [53, 54]. To prevent overaccumulation of FFOOH, cells sequester free PUFAs into the hydrophobic core of a lipid‐protein complex assembled by Fas‐associated factor 1 (FAF1), a cytosolic protein [55, 56]. In cells deficient in *FAF1*, FFOOH rapidly accumulated upon treatment with physiological levels of PUFAs, causing ferroptosis even in the absence of compounds that block GPX4 activity, which were required to trigger ferroptosis of the wild type (WT) cells. The FAF1‐mediated protection against ferroptosis is physiologically relevant, as mice with liver‐specific knockout of *Faf1* developed acute liver injury upon consuming a diet enriched in PUFAs [55, 56]. Thus, although ferroptosis is caused by PUFA‐PL peroxidation, preventing free fatty acid peroxidation also protects cells from ferroptosis [55, 56].

### 3.2 Propagation of Ferroptosis Across Cell Populations

Among all the cell death pathways characterized so far, ferroptosis is unique in that the cell death originating from a few cells can be propagated to cells at a distance through a wave‐like pattern [57, 58, 59, 60]. Previous reports have documented robust spatial propagation of cell death likely caused by ferroptosis over long distances in tissues or cell populations [57, 58, 59, 60]. However, the mechanism behind this propagation remains incompletely understood. Current models include diffusion‐based coupling, transport of peroxidized lipid species or related molecules, and contact‐dependent modes of intercellular spread. The relative contribution of these mechanisms is likely context‐dependent and remains an active area of investigation [57, 59, 61]. Understanding how ferroptosis is propagated may open opportunities to limit tissue damage in diseases caused by ferroptosis [57, 58, 59, 60, 61].

## 4 Tissue‐Specific Sensitivities: Lipid Metabolism as a Determinant

Although ferroptosis is defined by a common biochemical reaction, its susceptibility varies dramatically among tissues and cell types. Instead of the ferroptotic machinery itself, this diversity arises from unique metabolic reactions that shape distinct lipid composition, antioxidant defense, and iron homeostasis in different tissues. What varies, therefore, is not the existence of the ferroptotic machinery, but the metabolic context that determines how easily lipid peroxidation can be initiated, amplified, and contained [62, 63]. In this Review, we focus on the brain, liver, and testis/sperm as illustrative examples rather than an exhaustive survey, as these tissues represent distinct biologically informative configurations of ferroptosis vulnerability.

### 4.1 Brain

The brain is a lipid‐rich organ, with lipids comprising more than half of its dry mass [64, 65]. PLs are a major class of brain lipids, and they are remarkably enriched in PUFA acyl chains such as DHA and AA [66, 67]. These fatty acyl chains are indispensable for synaptic function, membrane excitability/dynamics, and lipid‐mediated signaling [68]. This biochemical advantage comes with an inherent liability: PUFA‐rich PLs provide abundant bis‐allylic sites that sustain radical chain reactions, thereby increasing vulnerability to lipid peroxidation [69]. Combined with high oxidative metabolism (the brain consumes a disproportionate fraction of systemic oxygen) and the limited regenerative capacity of adult central nervous system (CNS) neurons, neurons operate with a narrow margin for error when lipid peroxidation is engaged [70, 71]. It is therefore unsurprising that the nervous system has evolved layered defenses to keep PUFA peroxidation below the ferroptotic threshold. As such, mutations in genes involved in ferroptosis defense in humans can facilitate the development of neurodegenerative diseases.

In humans, a missense mutation of GPX4 that disrupts its interaction with membranes causes early‐onset neurodegeneration [72]. In adult mice, *Gpx4* ablation caused prominent mitochondrial damage together with neuronal loss and gliosis in the brains [73]. Conditional Gpx4 deletion in forebrain neurons triggered rapid neurodegeneration and cognitive impairment, and this phenotype was mitigated by the ferroptosis inhibitor liproxstatin‐1 [74], a compound originally identified to suppress pathology caused by Gpx4 inactivation in vivo [3]. This neurodegenerative phenotype was accompanied by increased production of oxidized phosphatidylethanolamine (PE) species, the production of which correlates with ferroptosis [16]. Conversely, GPX4 overexpression is sufficient to ameliorate cognitive impairment and reduce neurodegeneration in a genetic Alzheimer's disease (AD) mouse model [75]. These observations demonstrate the importance of GPX4 in protecting neurons from ferroptosis and support the view that failure of this defense may contribute to neuronal injury in certain neurodegenerative diseases.

ApoE4, a variant of human apoE, is the strongest genetic risk factor for late‐onset Alzheimer's disease [76, 77], possibly by weakening apoE‐dependent lipid‐handling pathways that help neurons cope with peroxidized lipids. Hyperactive neurons, through their intensive consumption of oxygen, drive lipid peroxidation. These peroxidized PUFAs are loaded onto apoE to form brain‐derived lipoprotein particles, which serve as a vehicle to transport these peroxidized PUFAs from neurons to astrocytes for degradation [78]. Interestingly, the lipid loading capacity is different among various isoforms of apoE, with apoE4 being the least efficient in lipid loading [79]. Consequently, mice expressing human apoE4 were more prone to lipid peroxidation in their neurons [80]. It will be interesting to determine whether the failure to protect neurons from ferroptosis is the mechanism through which apoE4 promotes neurodegenerative diseases.

### 4.2 Liver

In contrast to PUFA‐rich membranes in brains, the sensitivity to ferroptosis in livers is caused by the convergence of various metabolic reactions. Hepatocytes continuously synthesize, remodel, uptake, and export lipids, while simultaneously storing excess iron as ferritin and releasing it as needed. This characteristic creates a setting in which ferroptosis can be readily triggered under conditions in which iron‐catalyzed PUFA peroxidation overwhelms the ferroptotic defense mechanisms to cause liver diseases.

The best studied liver disease caused by ferroptosis is alcoholic liver disease (ALD). Overconsumption of alcohol leads to reactions favoring ferroptosis, such as increased hepatic iron load [81], elevated hepatic production of superoxide anion radicals [82], and higher hepatic PUFA levels [83]. Concurrently, alcohol feeding reduces levels of vitamin E and glutathione that protect hepatocytes from ferroptosis [84, 85]. Thus, excess alcohol consumption makes hepatocytes vulnerable to ferroptosis. Indeed, using the National Institute on Alcohol Abuse and Alcoholism (NIAAA) mouse model, the most extensively used animal model for ALD [86], it was demonstrated that hepatic injury under this condition was primarily caused by ferroptosis [87, 88]. A recent study demonstrated that feeding mice with alcohol through a diet enriched in PUFAs further exaggerated ferroptosis of hepatocytes. Under these circumstances, it took only 11 days of alcohol feeding for mice to develop advanced ALD marked by liver fibrosis [83]. In addition to ALD, ferroptosis also contributes to hepatic damage in metabolic dysfunction‐associated fatty liver disease (MAFLD), a more prevalent chronic liver disease, although the mechanism through which ferroptosis is stimulated under this circumstance is less clear [87, 89, 90, 91]. Importantly, “iron load” might be an oversimplified term from the perspective of ferroptosis. Rather than total tissue iron content, the availability of labile Fe(II) pools that are active in catalyzing PL peroxidation is more important in driving ferroptosis.

Besides dietary factors, genetic backgrounds may further shape how lipids are metabolized in the liver and thus determine ferroptotic sensitivity. For example, PNPLA3(I148M) is strongly associated with advanced ALD and MAFLD in humans [92, 93, 94]. Recent work supports a model in which PNPLA3(I148M) rewires flux of hepatic PUFAs from triglycerides (TGs) to PLs [95]. Similarly, a genetic variant in an intron of *MBOAT7*, which is involved in the synthesis of AA‐PLs, also increases the risk of developing these fatty liver diseases [96]. Considering that the sensitivity of cells to ferroptosis is determined by their levels of polyunsaturated PLs, it will be interesting to determine whether these genetic variants increase the risk of advanced ALD/MAFLD by sensitizing hepatocytes to ferroptosis.

### 4.3 Testis and Sperm

Spermatozoa represent an extreme case of enrichment of polyunsaturated PLs without adequate protection of ferroptosis by GPX4. Their membranes are highly enriched in long‐chain PUFAs, particularly DHA and docosapentaenoic acid (DPA), which enable the flexibility and curvature dynamics required for motility and fertilization [97, 98, 99]. Yet unlike other tissues, the functions of GPX4 in sperm are not only enzymatic, but architectural [100, 101, 102]. In addition to the canonical cytosolic GPX4, testis expresses two other isoforms of GPX4 caused by alternative splicing: the mitochondrial isoform (mGPX4), the prevailing product in male germ cells, and nuclear GPX4 (nGPX4) localized to the nuclear matrix in haploid germ cells.

As spermatids mature, there is a virtual absence of GSH in developing sperm [103]; in parallel, mGPX4 undergoes oxidative crosslinking and transitions from a PL hydroperoxidase into a structural component of the mitochondrial sheath/capsule [100, 101, 102], and nGPX4 becomes a structural component of chromatin by physically associating with protamines [102, 104, 105]. 99, 100Genetic evidence underscores this specialization. Targeted disruption of nGpx4 or mGpx4 did not impair embryonic development, yet produced sharply defined sperm defects: nGpx4 deficiency compromised chromatin condensation, whereas loss of germ‐cell GPX4 activity disrupted mitochondrial sheath/capsule organization and resulted in male infertility [100, 106, 107]. Consequently, mature sperm carry membranes rich in PUFA‐containing PLs, but with a markedly reduced capacity for GPX4‐dependent detoxification at the very stage when oxidative insults can be most consequential.[99, 100]These features define sperm as a cell type with an intrinsically low tolerance for lipid peroxidation, a setting in which ferroptosis is mechanistically plausible and biologically impactful [1, 101].

This configuration raises an unresolved but important question: how do sperm avoid catastrophic peroxidation during their lifespan in the male and female reproductive tracts? The prevailing implication is that other ferroptosis‐protecting mechanisms must compensate for the attenuated GPX4 activity in mature sperm [101, 108]. One potential such mechanism is FAF1‐mediated inhibition of ferroptosis. FAF1 sequesters free PUFAs in a cytosolic lipid–protein complex, thereby protecting cells from ferroptosis both in vitro and in vivo [56]. Inasmuch as FAF1 shows the highest levels of expression in testis and male germ cells, it will be interesting to determine whether this protein indeed plays a critical role in preventing ferroptosis of mature sperm. Nevertheless, even if the compensatory mechanisms do exist, they are not expected to be as robust as GPX4 in protecting against ferroptosis. Thus, the sensitivity to ferroptosis may limit the lifespan of sperm to ensure successful fertilization [101, 109]. At present, however, this remains a hypothesis, intriguing, but not established mechanistically.

## 5 Therapeutic Strategies to Treat Diseases Caused by Ferroptosis

Studies over the past decades have revealed the molecular pathways governing ferroptosis and the strategies to inhibit ferroptosis in cells cultured in vitro. However, owing to the lack of a ferroptosis marker that can be readily applied to patient samples, identification of diseases caused by ferroptosis has been challenging until the recent identification of hyperoxidized peroxiredoxin 3 (SO_2/3_‐PRDX3) as a potential marker for ferroptosis [87]. Even with such advances, developing therapeutic strategies to treat diseases caused by ferroptosis remains challenging, as the approaches that inhibit ferroptosis in vitro may not be directly translatable to clinical application.

### 5.1 Identification of Diseases Caused by Ferroptosis

A practical obstacle in translating ferroptosis biology to clinical application is the lack of a marker to identify diseases that are caused by ferroptosis. Historically, ferroptosis has often been inferred from bulk readouts of lipid peroxidation, such as malondialdehyde (MDA) and 4‐hydroxynonenal (4‐HNE) [16, 110]. These markers are useful indicators of oxidative lipid damage but are not specific for ferroptosis, as they arise from PUFA peroxidation regardless of whether the PUFAs are incorporated into PLs. Consequently, they may also increase in other forms of cell injury associated with oxidative stress, such as apoptosis [110, 111].

To improve tissue‐level detection, we recently identified SO_2/3_‐PRDX3 as a promising marker that shows a strong association with ferroptosis in systems examined to date [87]. PRDX3 is a thioredoxin‐dependent peroxidase localized to mitochondria [112]. Under ferroptotic stress, mitochondrial lipid hydroperoxides induce selective hyperoxidation of the catalytic cysteine of PRDX3 to sulfinic/sulfonic acid derivatives [87, 113, 114]. Importantly, SO_2/3_‐PRDX3 was robustly induced by ferroptosis but not other cell death pathways or mitochondrial damage unrelated to ferroptosis, supporting its use as a potential ferroptosis marker [87]. Hyperoxidized PRDX3 can be detected by immunoblotting of tissue lysates or immunostaining of tissue sections with 5H7c, a rabbit monoclonal antibody recognizing this ferroptosis marker [87, 88]. These assays provide a practical entry point for the detection of ferroptotic stress in tissues.

### 5.2 Challenge in Treating Diseases Caused by Ferroptosis: Strategies Effective In Vitro Do Not Guarantee Benefit In Vivo

Another challenge in developing strategies to treat diseases caused by ferroptosis is that the well‐established dogma on ferroptosis inhibition in vitro may not be applicable in vivo. This point is illustrated by the importance of ACSL4 on ferroptosis. In cells cultured in vitro, ACSL4 is required for the incorporation of long‐chain ω‐6 PUFAs into PLs, thereby sensitizing cells to ferroptosis. As a result, knocking out *ACSL4* prevents compounds that inhibit GPX4 from inducing ferroptosis [15]. However, in vivo, this strategy does not always translate into ferroptosis protection. For example, liver parenchymal cell–specific *Acsl4* deletion showed no significant improvement in the onset or progression of metabolic dysfunction‐associated steatotic liver disease (MASLD), a disease in which ferroptosis has been implicated [115]. A possible explanation for this discrepancy is that ACSL4 is required for ferroptosis induced through inhibition of GPX4, a condition employed in vitro that is unlikely to be the mechanism through which ferroptosis is stimulated in vivo. Indeed, knocking out *ACSL4* failed to rescue cells from ferroptosis induced by tert‐butyl hydroperoxide, an organic hydroperoxide [116].

In addition to molecular pathways, compounds including iron chelators that are remarkably effective in blocking ferroptosis in cells cultured in vitro [1, 3] have their limitations in vivo. In a multicenter, randomized, double‐blind phase 2 FAIRPARK‐II trial of 372 newly diagnosed Parkinson's disease patients who had not received dopaminergic therapy, the iron chelator deferiprone led to clinical worsening: 22.0% of participants in the deferiprone group required initiation of levodopa therapy owing to symptom progression, compared with 2.7% in the placebo group, and clinical rating‐scale scores were significantly worse at the end of the 36‐week treatment period [117, 118]. Neuroimaging in a subgroup confirmed that deferiprone reduced iron levels in the substantia nigra, indicating target engagement, yet this did not translate into clinical benefit [117, 118]. The FAIRPARK‐II primary report and accompanying commentary collectively underscore the translational dilemma: reducing an “iron metric” can coexist with deterioration in clinical outcomes [118]. Similar to Parkinson's disease, a recent clinical trial to treat Alzheimer's disease with deferiprone also ended up with disappointing clinical outcomes [119].

These results do not necessarily negate a pathogenic role for iron‐catalyzed lipid oxidation in neurodegeneration. Rather, they emphasize a basic constraint: in vivo, iron is not merely a pathogenic catalyst; it is also a required physiological resource. For instance, dopamine biosynthesis depends on tyrosine hydroxylase, a non‐heme iron‐dependent enzyme, making dopaminergic systems particularly sensitive to iron perturbation [120, 121]. Excessive or poorly timed iron depletion therefore risks iron deficiency in pathways essential for neuronal functions, thereby worsening clinical outcomes despite improving an iron‐burden readout [118, 120, 121, 122]. Accordingly, the main translational bottleneck for ferroptosis inhibitors is often not whether ferroptosis can be suppressed, but whether it can be suppressed at the right time, in the right tissue, and to the right degree (Box 1), without compromising essential iron‐dependent physiology [118, 122].

### 5.3 Nutritional Intervention as an Effective Strategy to Manage Liver Diseases Caused by Ferroptosis

Despite the challenges described above, nutritional intervention has emerged as a cost‐effective approach to managing diseases caused by ferroptosis. Diet shapes membrane lipid composition and therefore offers a physiological entry point to tune ferroptosis sensitivity [39, 69]. PUFAs are indispensable for membrane fluidity and signaling, yet PUFA‐PLs are also the key substrates that sustain lipid peroxidation [39, 62, 69]. In contrast, MUFAs, typified by OA, act as a substrate‐level buffer by diluting PUFA‐PLs within membranes. Thus, MUFA enrichment lowers peroxidation pressure of the lipid bilayer, thereby protecting cells from ferroptosis [39, 123]. This principle is particularly intuitive in explaining the benefit of the Mediterranean diet on the management of MAFLD. The cornerstone of the Mediterranean diet is olive oil, which is enriched in MUFAs, especially OA [124, 125]. In light of the mechanisms discussed above, OA is expected to suppress ferroptosis by promoting MUFA incorporation into membrane PLs, thereby reducing the effective PUFA‐PL substrate pool available for peroxidation [39]. Thus, the empirical observation that olive oil–based Mediterranean diet prevents the development of MAFLD is mechanistically aligned with an anti‐ferroptotic PL composition [126, 127].

The same mechanism may help to explain a long‐standing mystery regarding the Mediterranean diet, that is, alcohol consumption, a characteristic of the Mediterranean lifestyle, does not increase the risk of advanced ALD. Considering that alcohol induces hepatic damage through ferroptosis, olive oil, the source of OA in the Mediterranean diet, may reduce the severity of ferroptosis induced by alcohol, thereby preventing the development of advanced ALD. The benefit of olive oil in preventing advanced ALD was directly demonstrated through a mouse model of the disease in that mice fed alcohol through a diet based on olive oil failed to develop advanced ALD. In contrast, ∼30% of the mice fed with alcohol through a PUFA‐rich diet based on fish oil progressed to advanced ALD following the same procedure [83].

## 6 Perspective

Over the past decade, ferroptosis research has been driven largely by mechanistic discovery. These studies established ferroptosis as a form of regulated cell death driven by iron‐dependent PL peroxidation, and clarified a central principle: cellular susceptibility to ferroptosis is strongly shaped by membrane lipid composition, particularly the abundance of PUFA‐PLs, which serve as the substrates for chain‐propagating PL peroxidation. These mechanistic insights created a coherent framework linking lipid metabolism, redox control, and membrane integrity to ferroptotic death [1, 2, 15, 16, 128].

The field is now entering a new stage. A major bottleneck for translating ferroptosis biology into human disease has been the lack of a practical and specific marker that can be applied to tissues to detect ferroptotic cells. The identification of hyperoxidized PRDX3 (SO_2/3_‐PRDX3) provides a promising entry point for the detection of ferroptotic cells in tissues, partially addressing a major translational bottleneck; however, broader validation across disease contexts and human specimens remains necessary [87, 88]. These advances shift the translational question from “can ferroptosis be induced or blocked in cultured cells” to “where, when, and to what extent does ferroptosis occur in vivo,” and, critically, which disease phenotypes are causally driven by ferroptosis rather than indirect consequence of nonspecific oxidative damage [24, 83, 90, 129]. The possibility to map spatial and temporal resolution of ferroptosis in tissues should accelerate the development of treatments that modulate ferroptosis sensitivity through modification of dietary lipid composition, targeted control of PL remodeling, or reinforcement of endogenous anti‐peroxidation defenses, while minimizing systemic liabilities [5, 15, 16, 39].

A key challenge, however, is that strategies effective in vitro do not necessarily confer benefit in vivo. In cells cultured in vitro, ferroptosis is often triggered by acute pharmacologic inhibition of GPX4 or cystine import [1, 4]. In vivo, ferroptosis is more likely initiated by distinct upstream stresses, unfolding over different time scales, and occurring within complex tissue environments where iron, lipid trafficking, inflammation, and tissue repair programs are tightly coupled [2, 62]. Thus, genetic disruption of certain lipid remodeling enzymes or treatment with small‐molecule inhibitors that markedly inhibit ferroptosis in vitro might not function as effectively in vivo. Moreover, interventions that broadly perturb iron or lipid metabolism can carry physiological costs that offset anti‐ferroptotic benefits, particularly in organs with high metabolic demand or specialized functions [115, 117, 118, 119].

Progress in the next stage of ferroptosis research will therefore depend on integrating marker‐based pathology with mechanism‐based investigations in vivo [2, 87]. Priorities include defining the upstream triggers and cellular sources of lipid peroxides in tissues, determining the cell types that initiate and propagate ferroptosis signals, and establishing therapeutic windows in which ferroptosis can be suppressed without disrupting essential iron‐ and lipid‐dependent physiology [2, 62, 115, 117, 118, 119]. With these advances, ferroptosis research will progress beyond proof‐of‐principle inhibition in cultured cells toward mechanism‐guided, context‐specific interventions for human diseases driven by ferroptosis.

**Proposed translational framework: the “right time, right tissue, right degree” principle**

To apply ferroptosis‐targeted therapy in vivo, three variables should be considered explicitly. Right time: Define intervention windows using pathology based on tissue ferroptosis markers rather than symptom stage alone. Right tissue: Prioritize local or cell‐type‐enriched intervention strategies to minimize systemic perturbation of iron‐ and lipid‐dependent physiology. Right degree: Aim for partial suppression sufficient to lower ferroptotic stress below a damage threshold, rather than maximal inhibition such as broad iron depletion; Preservation of essential iron‐dependent functions should be evaluated as a co‐endpoint. This framework may help to translate robust anti‐ferroptotic activity discovered in vitro into clinical benefit in vivo.

## Conflicts of Interest

The authors declare no conflicts of interest.

## Data Availability

The authors have nothing to report.
